# Direct observation of multiband transport in magnonic Penrose quasicrystals via broadband and phase-resolved spectroscopy

**DOI:** 10.1126/sciadv.abg3771

**Published:** 2021-08-25

**Authors:** Sho Watanabe, Vinayak S. Bhat, Korbinian Baumgaertl, Mohammad Hamdi, Dirk Grundler

**Affiliations:** 1 School of Engineering, Institute of Materials, Laboratory of Nanoscale Magnetic Materials and Magnonics, École Polytechnique Fédérale de Lausanne, EPFL, 1015 Lausanne, Switzerland.; 2 International Research Centre MagTop, Institute of Physics, Polish Academy of Sciences, 02668 Warsaw, Poland.; 3 School of Engineering, Institute of Electrical and Micro Engineering, École Polytechnique Fédérale de Lausanne, EPFL, 1015 Lausanne, Switzerland.

## Abstract

Quasicrystals are aperiodically ordered structures with unconventional rotational symmetry. Their peculiar features have been explored in photonics to engineer bandgaps for light waves. Magnons (spin waves) are collective spin excitations in magnetically ordered materials enabling non–charge-based information transmission in nanoscale devices. Here, we report on a two-dimensional magnonic quasicrystal formed by aperiodically arranged nanotroughs in ferrimagnetic yttrium iron garnet. By phase-resolved spin wave imaging at gigahertz frequencies, multidirectional emission from a microwave antenna is evidenced, allowing for a quasicontinuous radial magnon distribution, not observed in reference measurements on a periodic magnonic crystal. We observe partial forbidden gaps, which are consistent with analytical calculations and indicate band formation as well as a modified magnon density of states due to backfolding at pseudo-Brillouin zone boundaries. The findings promise as-desired filters and magnonic waveguides reaching out in a multitude of directions of the aperiodic lattice.

## Teaser

Magnonic quasicrystals give rise to multidirectional spin wave emission and the formation of a pseudo-Brillouin zone.

## INTRODUCTION

Since the discovery of quasicrystals over three and a half decades ago (*1*), their aperiodicity and unconventional rotational symmetries combined with long-range order have baffled physicists and material scientists alike. Recent advances in nanofabrication techniques have allowed researchers to explore the effects of aperiodicity using the materials-by-design approach (*2*). The prime examples of this approach are the studies on artificial photonic crystals and plasmonic crystals (*3*, *4*) based on the 10-fold rotational symmetric Penrose tiling—a two-dimensional (2D) analogue of 3D quasicrystals (*5*, *6*). Such photonic quasicrystals offered a complete bandgap for light (*7*, *8*, *9*), whereas the observation of strong transmission peaks in artificial plasmonic quasicrystals refuted the conventional well-accepted view that periodicity is crucial for such observation (*4*).

Recently, insights on the effect of aperiodicity were gained in magnonic quasicrystals (MQCs) from the study of spin waves (SWs) (*10*–*12*). The bicomponent MQCs based on a Fibonacci sequence (1D) and Penrose tiling (2D) exhibited a multilevel structure of magnonic bandgaps in simulations (*11*, *13*). These gaps and SW propagation in 1D quasicrystals were evidenced using Brillouin light scattering (BLS) and x-ray microscopy techniques (*14*, *15*). Furthermore, worm-like nanochannels found in 2D antidot quasicrystals gave rise to an unprecedented demultiplexing process with microwaves that showed distinct advantages over demultiplexing process in photonics (*12*). In addition, the reprogrammability of artificial quasicrystals (*10*, *14*, *16*) promised a data writing process. This makes the study of MQCs timely from the perspective of nanomagnonics, which is an evolving branch in magnetism exploring SWs (*17*). On the nanoscale, magnon-based logic circuits are expected to transfer and process information efficiently (*18*).

One of the prominent limitations of earlier works is that they were based on ferromagnetic metals. Previous studies were done on materials for which their SW damping might have obscured further coherent SW backscattering effects like the formation of a complete magnonic bandgap. Such a gap is key for the creation of multidirectional magnonic waveguides and SW cavities inside a magnonic crystal (MC) by analogy with a photon cavity residing in a defect of a photonic crystal (*19*). Because of the low rotational symmetry, the formation of a complete magnonic bandgap is more challenging for MCs based on periodic lattices (*20*) than for quasicrystals, which can exhibit much higher rotational symmetry. The latter feature is advantageous also for multidirectional magnon emission in case of the magnonic grating coupler effect (*21*–*24*), which, however, has not yet been reported for 2D quasicrystals.

**Fig. 1:**
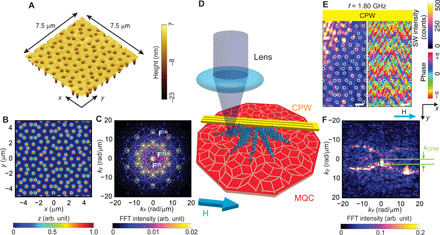
Omnidirectional SW emission in an aperiodic MQC. (**A**) Atomic force microscopy image of nanotroughs of a diameter *D* ∼ 300 nm etched into 100-nm-thick YIG at the vertices of a Penrose P3 tiling and (**B**) their virtual real and (**C**) reciprocal space representation. Green circles indicate reciprocal vectors. (**D**) Sketch of the BLS experiment showing an integrated CPW as an SW emitter on an MQC. (**E**) Spatial distribution of magnitude (left) and phase (right) of emitted SWs at *f* = 1.80 GHz in a field of 10 mT applied along the +*x* direction. Scale bars, 1 μm. Bright (dark) in the left image indicates large (small) spin-precessional cone angles. Hue scale in the right image represents the phase of SWs. White circles indicate positions of the nanotroughs. (**F**) Fourier transform of the data shown in (E). The horizontal lines highlight *k*_CPW_ ∼ (0,2.8 ± 0.4) rad/μm provided by the CPW at which the local maximum of SW intensity (bright) is found. The dumbbell-shaped iso-frequency contour indicates dipole-dominated SWs propagating in an omnidirectional manner. A geometrical misalignment of CPW with respect to MQC and scanning directions might induce its slight rotation in reciprocal space. FFT intensities of (C) and (F) are normalized to the maximum intensity.

**Fig. 2:**
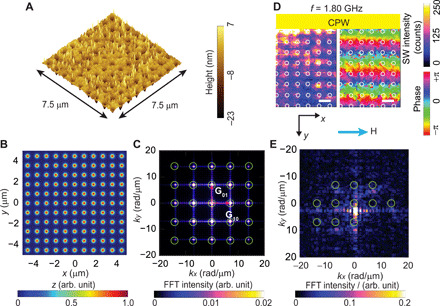
Multidirectional SW emission in a periodic MC. (**A**) Atomic force microscopy image of MC based on the square lattice of period *a* ∼ 900 nm, where nanotroughs of a diameter of *D* ∼ 300 nm were etched out from a YIG thin film. The virtual representation of the square lattice in (**B**) real space and (**C**) reciprocal space. Light green indicates the reciprocal vectors of the square lattice. (**D**) Spatial distribution of SWs in magnitude (left) and phase (right) at *f* = 1.80 GHz propagating in the +*y* direction in the MC. The field of 10 mT was applied along the CPW long axis (+*x* direction). Scale bars, 1 μm. Bright (dark) color in the left image indicates large (small) spin-precessional cone angles, and the hue scale in the right image represents the phase of SWs. The white circles indicate the positions of nanotroughs. Green circles indicate calculated Bragg spots of reciprocal lattice vectors for comparison. (**E**) SW intensity in reciprocal space after FFT on the SW images of (D). Reciprocal lattice vectors **G** are added to the SW mode excited with *k*_CPW_ ∼ (0,2.8 ± 0.6) rad/μm at *f* = 1.8 GHz by the CPW. The value of *k*_CPW_ is extracted from the local intensity maximum near (*k_x_*, *k_y_*) = (0,0). FFT intensity of (C) and (E) is normalized with respect to the maximum intensity.

Here, we investigate SW properties of MQCs. The MQCs were prepared from an yttrium-iron-garnet (YIG) thin film, a low-damping insulating ferrimagnet (*25*), by etching out circular nanotroughs at the vertices of Penrose P3 tiling (Fig. 1A). The SW damping in YIG is about 10^2^ smaller as compared to previously studied NiFe and CoFeB films, allowing us to explore further previously unidentified aperiodicity-related effects on band structure. The Penrose lattice (Fig. 1B), because of its self-similarity and long-range ordered nature, shows sharp peaks (Bragg peaks) in reciprocal space that define specific reciprocal vectors **F** (Fig. 1C). Subsequently, coplanar waveguides (CPWs) integrated on the MQC allowed us to excite and characterize SWs using spatially resolved inelastic (Brillouin) light scattering (BLS) and broadband SW spectroscopy techniques (Fig. 1D). We observed SWs propagating in diagonal directions (Fig. 1E), in contrast to reference periodic lattice of nanotroughs in YIG (see Fig. 2). The Fourier transformation of complex SW images obtained on the quasicrystalline arrangement of nanotroughs (Fig. 1F) gives strong indication that the SWs are emitted in a truly omnidirectional manner. This has not been reported from the study of conventional magnonic grating couplers based on periodic structures. Moreover, we observed features consistent with a 2D pseudo-Brillouin zone (p-BZ) for SWs and a modified magnonic density of states (DOS) reflecting partial magnonic bandgaps in the SW dispersion relation. Our experimental results suggest that a Penrose P3 tiling operates as a grating coupler inducing a manifold of both propagation directions and wavelengths. Such formation of magnonic minibands allows one to engineer magnonic waveguides by leaving out columns of nanoholes without the directional restrictions exhibiting distinct advantage over previously explored periodic nanohole lattices (*26*).

## RESULTS

### Detection and imaging omnidirectional SW emission

We first present BLS microscopy performed on the aperiodic Penrose P3 lattice (sample P3-MQC) (Fig. 1) and a reference periodic lattice (Fig. 2). Physical parameters and lattice characteristics are summarized in Table 1. BLS data were taken near the CPW by which we excited SWs, and an in-plane magnetic field *H* was applied parallel to the CPW and along the symmetry axis (*x* axis) of P3-MQC. Here, a bare CPW allows for the excitation of SWs that propagate along the *y* direction. Before discussing MQC data in detail, it is instructive to consider the SW imaging data taken on an MC (Fig. 2A), incorporating a translationally invariant square lattice of nanotroughs (sample SQ-MC).

**Table 1:** Parameters of prepared and investigated samples. The YIG films were 100 nm thick. The overall area of the samples was 52.8 μm × 50.2 μm. The CPW signal line width was 0.8 μm. Error bars of diameter *D* and depth *t* of nanotroughs indicate the 95% confidence interval of the diameters of five nanotroughs, which were chosen from the respective microscopy image at the center of the samples.

**Sample name**	**Equipment**	**Lattice**	**Type of lattice** **arrangement**	**Rotational** **symmetry in** ***k*-space**	**Characteristic** **length *a* (nm)**	**Diameter *D*** **(nm)**	**Depth *t* (nm)**
PF	VNA	–	–	–	–	–	0
SQ-MC	VNA	Square	Periodic	4	900	288 ± 10	21.7 ± 1.5
SQ-MC	BLS	Square	Periodic	4	900	272 ± 23	8.8 ± 2.4
P3-MQC	VNA/BLS	Penrose P3	Aperiodic	10	900	299 ± 18	23.7 ± 2.1

**Fig. 3:**
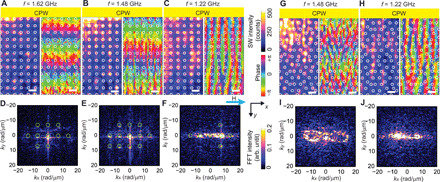
SW propagation in a periodic MC and an aperiodic MQC. Spatial distribution of SW magnitude (left) and phase (right) at (**A**) *f* = 1.62, (**B**) *f* = 1.48, and (**C**) *f* = 1.22 GHz, propagating in the +*y* direction in sample SQ-MC. The field of 10 mT was applied along the CPW long axis (+*y* direction). Scale bars, 1 μm. The bright (dark) color in the left image indicates large (small) spin-precessional cone angle, and a hue scale in the right image represents SW phase. SW intensity in reciprocal space after an FFT performed on the SW images taken at (**D**) *f* = 1.62, (**E**) *f* = 1.48, and (**F**) *f* = 1.22 GHz. Green circles indicate calculated Bragg spots of reciprocal lattice vectors for comparison. Spatial distribution of SW magnitude (left) and phase (right) at (**G**) *f* = 1.48 and (**H**) *f* = 1.22 GHz, propagating in the +*y* direction in sample P3-MQC. SW intensity in reciprocal space after an FFT performed on the SW images taken at (**I**) *f* = 1.48 and (**J**) *f* = 1.22 GHz. The wave vector **k**_CPW_ extracted from BLS data in reciprocal space amounts to (0,1.5 ± 0.4) rad/μm at *f* = 1.62 GHz in (D), (0,0.9 ± 0.4) rad/μm and (0,0.9 ± 0.5) rad/μm at *f* = 1.48 GHz in (E) and (I), respectively, and (0,0 ± 0.5) rad/μm and (0,0 ± 0.8) rad/μm at *f* = 1.22 GHz in (F) and (J), respectively. FFT intensities of (D) to (F), (I), and (J) are each normalized with respect to their maximum intensity.

Figure 2 (A to C) illustrates the periodic square lattice in both real and reciprocal space. The spatial distribution of SWs in SQ-MC at *f* = 1.80 GHz (Fig. 2D) shows wavefronts of SWs propagating parallel to the CPW. We analyzed the excited wave vectors of SWs for SQ-MC (Fig. 2E) by fast Fourier transformation (FFT) of complex-valued SW images. Here, the highest-intensity FFT peak close to (*k_x_*, *k_y_*) = (0,0) is observed at **k** = (*k_x_*, *k_y_*) ∼ (0,2.4) rad/μm. This wave vector is anticipated by the SW dispersion relation for Damon-Eschbach (DE) modes at *f* = 1.80 GHz in the unpatterned YIG thin film (fig. S1A), and the wave vector is attributed to the CPW [**k**_CPW_ = (0, *k*_CPW_)]. In addition to this mode, sharp peaks appear periodically with spacings of 7.0 rad/μm ∼ 2 π/(900 nm). The spacings correspond to reciprocal lattice vectors **G** of SQ-MC. That is, we observe SWs with wave vectors **k**_CPW_ + **G**, where **k**_CPW_ = (0, *k*_CPW_) is the wave vector imposed by the CPW at a given frequency. This set of peaks is consistent with the magnonic grating coupler effect (*21*). It evidences coherent scattering of SWs and the backfolding of the SW dispersion via reciprocal lattice vectors. In (*27*), it was argued that periodically modulated demagnetization fields induced by nanoholes in an MC served as scattering potentials for Bragg scattering of SWs. At *f* = 1.62 GHz (Fig. 3A), the wavefronts display a longer wavelength in the *y* direction compared with that in Fig. 2D. At *f* = 1.48 GHz (Fig. 3B), the wavelength has increased further and the wavefronts appear to be distorted. Last, at *f* = 1.22 GHz (Fig. 3C), the wavelength in the *y* direction can no longer be extracted from the field of view (λ ≥ 10 μm) and the wavefronts appear to be subdivided into channels extending parallel to the *y* direction. Subsequently, the corresponding FFT results (Fig. 3, D to F) show that beyond the pronounced mode with (*k_x_*, *k_y_*) = (0, *k*_CPW_), the SW patterns contain excitations with reciprocal lattice vectors $\left(k_{x},k_{y})=(\pm p\cdot \frac{2\pi }{a},k_{\text{CPW}}\pm p\cdot \frac{2\pi }{a}\right)$, with *p* = 0,1. In Fig. 3 (D and E), the wavelengths of SWs correspond to discrete *k* values in *x* and *y* directions. At low frequency *f* = 1.22 GHz in Fig. 3F, FFT peaks are found near (*k_x_*, *k_y_*) ∼ (±7.0,0) rad/μm instead of (0,0) rad/μm. Here, SWs were elongated along the +*y* direction underneath nanotroughs, and standing SWs were confined in the *x* direction, which results in the formation of SW nanochannels (*28*). The SW phase within neighboring nanochannels is almost identical, while regions with large and small spin-precessional amplitudes are out of phase with each other.

The real-space SW map in the quasicrystalline structure (Fig. 1E) shows irregular SW wavefronts compared to SQ-MC at *f* = 1.80 GHz. The corresponding FFT image (Fig. 1F) contains a dumbbell-shaped iso-frequency contour in *k*-space. Unexpectedly, such iso-frequency contour agrees with the anisotropic characteristics (dispersion relations) of SWs in an unpatterned YIG film (see figs. S1B and S2A). The data indicate that the microwave current in the CPW induces an inhomogeneous radio frequency field *h*_rf_ that emits SWs into almost any direction at *f*. Reciprocal lattice vectors are not defined for an aperiodic Penrose P3 lattice; still, Bragg peaks are seen in diffraction pattern of a quasicrystal due to its self-similar and long-range ordered properties (Materials and Methods and Fig. 1C). The corresponding reciprocal vectors **F** densely fill out the reciprocal space (*8*). Such reciprocal vectors might provide the relevant Fourier components for *h*_rf_ to excite SWs in the nearly omnidirectional manner at fixed *f* as evidenced by the observed iso-frequency contour in *k*-space. This functionality is valid for a single frequency and thereby different from the previously reported grating couplers based on periodic lattices for which quasi-omnidirectional emission was claimed for SWs of different frequencies excited within a broad frequency band (*21*).

The wavelengths of SWs are found to increase in P3-MQC (Fig. 3, G and H, for *f* = 1.48 and *f* = 1.22 GHz, respectively) when the excitation frequency is reduced; this trend is similar to the SWs excited in SQ-MC. The extension of the dumbbell-shaped iso-frequency contour at *f* = 1.48 GHz for P3-MQC (Fig. 3I and fig. S2B) is smaller, because the anisotropy of SWs at *f* = 1.48 GHz involves smaller wave vectors compared to *f* = 1.80 GHz. The dumbbell-shaped contour is now closed (Fig. 3I), as the maximum wave vector *k* resides in the sensitivity range of the BLS.

The FFT maps at *f* = 1.22 GHz for SQ-MC (Fig. 3F) and P3-MQC (Fig. 3J) show wide intensity distributions along the *k_x_* direction. These indicate that in real space, the SW excitations are confined along the *x* direction, i.e., in both samples, nanochannels are formed and extend in the *y* direction (orthogonal to **H**). The nanochannel formation is due to the inhomogeneous internal field, which takes locally small values because of the demagnetization effect of the nanotroughs, and resonant spin precession occurs at correspondingly small excitation frequency. The nanochannels in P3-MQC appear to be irregular, consistent with (*12*). In the following, we present broadband SW spectroscopy based on a vector network analyzer (VNA) to explore the frequency dependence of SW absorption and transmission in P3-MQC in detail and compare the results with SQ-MC and a plane film.

### Manifold SW emission

**Fig. 4:**
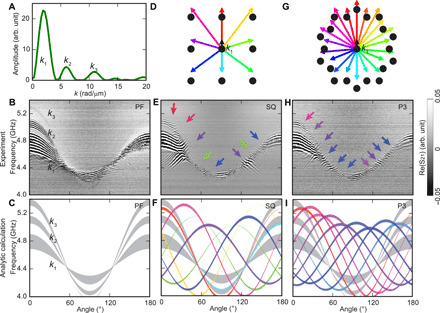
Angular-dependent SW spectra in transmission configuration. (**A**) Excitation spectrum by Fourier transformation of the CPW microwave magnetic field in the *y* direction. (**B**) Experimental and (**C**) calculated angular-dependent SW spectra of PF at a field of μ_0_*H*_0_ = 93 ± 3 mT. (**D**) Vectors of grating coupler–induced modes **k**_1_ + **G***_ij_* shown in different colors in reciprocal space of the SQ lattice (black dots). (**E**) Experimental and (**F**) calculated spectra of the SQ lattice. Gray bands represent SW branches of **k**_**1**_, **k**_**2**_, and **k**_**3**_. Colored lines indicate the backfolded SW modes. Considering the full width at half maximum Δ*k_i_* for peaks labeled by *k_i_* (with *i* = 1,2,3) in (A), the width of gray bands expresses the frequency band corresponding to *k_i_* − Δ*k_i_*/2 ≤ *k_i_* ≤ *k_i_* + Δ*k_i_*/2. The thickness of the colored lines represents the amplitude of propagating SWs considering their nonreciprocity. Arrows indicate the modified SW spectra. (**G**) Reciprocal lattice points of the P3-MQC lattice. (**H**) Experimental and (**I**) calculated SW spectra of P3-MQC encoded and labeled in a consistent way with the SQ data. Colors in (E) and (F) and (H) and (I) highlight different SW resonance modes attributed to wave vectors defined in (D) and (G), respectively. In (B), (E), and (H), black and white represent the phase variation of SWs due to wave vector–dependent phase accumulation between two CPWs. Pronounced oscillations are labeled by **k**_**1**_, **k**_**2**_, and **k**_**3**_ in (B), consistent with the excitation spectrum of the CPW shown in (A).

We conducted angular-dependent SW spectroscopy in a rotating in-plane field *H* for the broadband characterization of SW properties in P3-MQC (Fig. 4). The field value of 90 mT was applied at various in-plane angles θ between the field direction and the CPW long axis. The SW resonances were determined using scattering parameters (S parameters) in absorption and transmission configuration using a VNA (see Materials and Methods). In the transmission configuration, two CPWs were used: one for excitation and one for detection of propagating SWs. The propagating SWs induced microwave voltages (S_21_), which, in real and imaginary part, oscillated as a function of frequency due to the phase accumulated by SWs between the CPWs. The period ∆*f* of the oscillating S_21_ signal scales linearly with the group velocity *v_g_* according to

$$v_{g}=\Delta f\cdot s$$ (1)

where *s* represents the distance between the two signal lines of the CPWs (*29*). The most prominent excitation for the CPWs used in this work is centered around *k*_1_ = 1.92 rad/μm (Fig. 4A). The second and third most prominent excitations of a bare CPW occur near *k*_2_ and *k*_3_, respectively. In the following, we present SW spectroscopy data taken by the VNA on three different samples: (i) an unpatterned YIG thin film (Fig. 4, B and C), (ii) the YIG film containing a periodic lattice (SQ-MC) of nanotroughs (Fig. 4, D to F), and (iii) quasi-crystalline lattice (P3-MQC) of nanotroughs (Fig. 4, G to I). All the three samples were obtained from the same wafer.

The S_21_ signals obtained on the plane YIG film (PF) are displayed in Fig. 4B. The oscillating S_21_ signals of propagating SWs appear in three pronounced frequency bands reflecting the wave vector distribution of the CPW around **k**_1_, **k**_2_, and **k**_3_. The signals are most pronounced near θ = 0°, and the bands reside at the lowest frequency near θ = 90°. The observed SW propagation modes follow the angular dispersion expected for dipole dominated SWs in a plane film. In Fig. 4C, we show analytically calculated SW spectra considering the excitation spectrum of CPWs (Fig. 4A) and the dispersion relation of the YIG thin film (*30*). Resonance frequencies of the prominent modes supported by the CPW follow a sinusoidal-like angular dependence due to the anisotropic dispersion relations of SWs. Oscillations in S_21_ vanish near θ ∼ 55° and 125°, indicating a group velocity close to zero. Correspondingly, the decay length *l_d_* = *v_g_*τ is small (τ is the lifetime of SWs), and therefore, the amplitude of S_21_ measured at the detector CPW is low. SWs with wave vectors **k**_1_, **k**_2_, and **k**_3_ are weak at θ = 180° due to the nonreciprocity of the DE-type SWs in a YIG film (*25*). The nonreciprocity parameter κ is defined in (*31*) as the ratio κ = *A*_0_(+*H*)/*A*_0_(−*H*) of S_21_ oscillation amplitudes *A*_0_(*H*) for positive and negative fields *H*. The maximum κ amounts to κ = *A*_0_(+90 mT)/*A*_0_(−90 mT) = 25 at *f* = 4.8 GHz in the unpatterned YIG plane film.

Considering previous studies on periodic antidot lattices and the magnonic grating coupler effect (*21*), we show in Fig. 4D relevant wave vectors (colored arrows) that are excited due to backfolding according to **k**_1_ + **G***_ij_* [**k**_1_ is the wave vector provided by the CPW (Fig. 4A) and *i*, *j* = 0, ±1]. We detect more complex SW spectra on SQ-MC (Fig. 4E) compared to PF (Fig. 4B). Oscillating SW propagation signals in sample SQ-MC appear even at θ ∼ 125° (purple arrow), where the vanishing group velocity in PF prohibited a propagation signal. These additional propagation signals highlight the modified SW wave vectors due to the grating coupler effect. The colored arrows in Fig. 4E indicate modifications in SW spectra due to wave vectors displayed in Fig. 4D in the corresponding colors. Figure 4F displays the calculated angular dependencies of backfolded SW modes using a color code consistent with Fig. 4D, and we compare these with the **k***_i_* modes in the plane film (gray colored lines). The width of a line related to the grating coupler effect (colored line) represents the expected SW amplitude considering the nonreciprocity of SWs. At the intersections of **k**_1_ and **k**_2_ modes (gray) with the backfolded modes, angular-dependent SW propagation signals are expected to be modified strongly as observed in the experiments. Note that some backfolded modes such as **k**_1_ + **G**_0(−1)_, **k**_1_ + **G**_1(−1)_, and **k**_1_ + **G**_(−1)(−1)_ do not reach the detector CPW due to the propagation direction opposite to **k**_1_. However, the CPW also offers −**k**_1_, and modes like −**k**_1_ − **G***_ij_* can be detected. Resonance frequencies of **k**_1_ + **G***_ij_* and −**k**_1_ − **G***_ij_* are identical.

In Fig. 4G, we display the Bragg peaks (black circles) and color-coded vectors, which represent **k**_1_ + **F** expected for P3-MQC. The Bragg peaks exhibit a manifold rotational symmetry, and vectors **F** are hence numerous. We focus on basic reciprocal vectors **F**^(1)^ and the second prominent reciprocal vectors **F**^(2)^ illustrated using differently colored arrows (compare Fig. 1C, and see Materials and Methods). Similarly colored arrows are used in Fig. 4H to highlight where SW modes **k**_1_ + **F** intersect with the **k**_1_ branch. Analytic calculations of a large set of SW modes corresponding to **k**_1_ + **F**, where **F** are different reciprocal vectors **F**^(1)^ and **F**^(2)^, are shown in Fig. 4I. Note that there are many additional reciprocal vectors **F**, of which the magnitudes are smaller than the basic reciprocal vectors **F**^(1)^ (Materials and Methods) (*8*), and therefore, more backfolded modes are possible than we indicate in Fig. 4I. The detailed inspection of the data shown in Fig. 4H underlines that the aperiodic quasi-crystalline lattice allows one to excite SWs propagating in many more directions than the periodic SQ-MC, which exhibits a lower rotational symmetry compared to P3-MQC.

### Formation of magnonic band structure in MQC

**Fig. 5:**
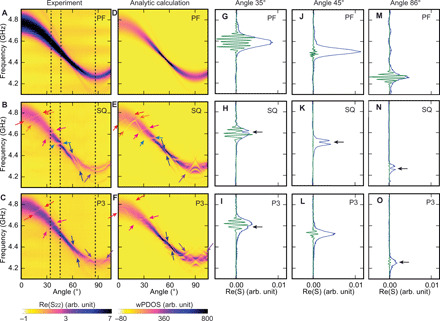
Angular-dependent SW spectra in reflection configuration. Angular-dependent SW spectra of (**A**) PF, (**B**) SQ-MC, and (**C**) P3-MQC in reflection geometry obtained with an applied field μ_0_*H* = 90 mT. Dark (bright) represents the SW absorption (background). Colored arrows indicate the modified SW absorption. Angular-dependent wPDOS for SWs of (**D**) PF, (**E**) SQ-MC, and (**F**) P3-MQC. Dark (bright) represents the high (low) value of wPDOS. Colored arrows represent the modified DOS corresponding to the forbidden frequency gaps in (B) and (C). Line cuts of SW spectra of PF (**G**, **J**, and **M**), SQ-MC (**H**, **K**, and **N**), and P3-MQC (**I**, **L**, and **O**) at an angle of 35° (G to I), 45° (J to L), and 86° (M to O). Blue (green) line represents S parameter in reflection (transmission) configuration. The amplitude of transmission spectra is multiplied by two to enhance the modification of the SW characteristics.

In the following, we present absorption spectra. The absorption strength is known to scale with the frequency-dependent magnon DOS. Figure 5 (A to C) displays measured SW absorption spectra of PF, SQ-MC, and P3-MQC, respectively (dark indicates large absorption). The overall sinusoidal characteristics of the dark bands of excited SWs are consistent with SWs expected for the PF. We find specific narrow regions for SQ-MC and P3-MQC (colored arrows in Fig. 5, B and C), where the signal strength is reduced or vanishes. We attribute these features to forbidden frequency gaps in SQ-MC and P3-MQC, which are not present in PF. Note that the modifications in the SW absorption spectra of SQ-MC and P3-MQC are different.

To understand the origin of the forbidden frequency gaps, the DOS of SWs is calculated from the SW dispersion relation for each sample. Here, the oscillator strength related to the projection of magnon eigenfunction and the excitation spectrum of the magnetic field *h*_rf_ is considered (see Materials and Methods). The angular-dependent weighted projected DOS (wPDOS) for PF is shown in Fig. 5D. At θ ∼ 55°, the wPDOS is high, as the slope of the SW dispersion relation in the dipolar regime is almost flat. The large wPDOS is consistent with the fact that the group velocity *v_g_* at θ ∼ 55° is almost zero, consistent with the results discussed for Fig. 4B.

Gallardo *et al.* considered the plane-wave method (*32*) for calculating the dynamic response and bandgaps of periodically modulated magnetic materials (*33*, *34*). We applied this method to our surface-modulated SQ-MC (see Materials and Methods). Figure 5E shows the wPDOS of SWs in SQ-MC when we consider the reciprocal lattice vectors **G***_ij_*, where *i*, *j* = 0, ±1, ±2, ±3 for the basis set in magnon band calculations. Modifications of the DOS are indicated by arrows. These changes are traced back to the partial bandgap openings in the SW dispersion due to the formation of BZs (see Materials and Methods). The calculated modifications are similar to the experimental results (displayed on the left), which indicates that the forbidden gaps observed in the experiments are derived from the backfolding effect and miniband formation in the periodically modulated MC.

Aperiodically arranged nanostructures do not have Brillouin zones because reciprocal lattice vectors **G** do not exist in quasicrystals. However, one can still choose the reciprocal vectors **F** of quasicrystals and assume corresponding backfolding of the SW dispersion (*33*, *34*). For our calculation, we consider reciprocal vectors **F**^(1)^, **F**^(2)^, and **F**^(3)^ for the basis set. In the backward volume wave (BVW) regime near θ ∼ 90°, the modification of the wPDOS is pronounced (Fig. 5F). This is because the original SW dispersion relation of BVW is relatively flat, and thus, backfolded SW dispersion relations interact with the original SW dispersion strongly (*34*). Consistent modifications are observed in the experiments on P3-MQC. The qualitative agreement indicates the formation of a p-BZ in a real MQC, which has not been reported before. The resolved p-BZ is defined by the basic reciprocal vectors **F**^(1)^ shown in Fig. 1C (*8*). The purple arrows indicate the angle-dependent modifications of SW absorption in the BVW regime.

Figure 5 (G to I) shows the line spectra of SW absorption and propagation signals at θ = 35° for PF, SQ-MC, and P3-MQC, respectively. The strong oscillation of Re(S_21_) due to SW propagation between emitter and detector CPW is detected at the frequency for which the SW absorption peaks in sample PF (Fig. 5G) are large. According to Eq. 1, the group velocity is 0.30 km/s. In sample SQ-MC (Fig. 5H), mode **k**_1_ and a backfolded mode **k**_1_ + **G** coexist in the same frequency regime. Here, a dip is observed in the SW absorption spectra at *f* = 4.6 GHz, with a width of about 20 MHz. The oscillation signal of SW propagation at this frequency is modified as well. We attribute this modification to the partial gap opening of the magnonic band structure of SQ-MC. The group velocity of SWs for sample SQ-MC below and above *f* = 4.6 GHz is 0.26 and 0.36 km/s, respectively, which indicates that different modes dominate the SW propagation above and below the frequency. A similar type of modification is also seen in sample P3-MQC (Fig. 5I).

At θ = 45°, observed modifications are of a different kind. In Fig. 5J, we report a strong absorption peak for sample PF, but the amplitude of SW propagation is very small due to the low group velocity of 0.17 km/s. For SQ-MC (Fig. 5K), a clear dip occurs in the absorption spectrum. The dip is more than half of the SW absorption strength. The SW propagation appears to be completely suppressed, most likely due to a pronounced gap opening in the SW band. For sample P3-MQC (Fig. 5L), the measured absorption peak is featureless, and contrary to PF and SQ-MC, a relatively strong SW propagation signal with a group velocity of 0.32 km/s is detected. We attribute this observation to backfolded modes forming an allowed miniband with an improved group velocity. At θ = 86°, BVW-SWs with a group velocity of 0.28 km/s were emitted in PF. Similar to the angle 45°, SW absorption is suppressed at *f* = 4.26 GHz in sample SQ-MC. At the same frequency, a slight modification of SW absorption is detected in sample P3-MQC. Here, the SW group velocity amounts to 0.3 km/s. Note that sample SQ-MC shows a very similar group velocity of 0.31 km/s above the frequency of *f* = 4.26 GHz, at which the absorption is suppressed.

**Fig. 6:**
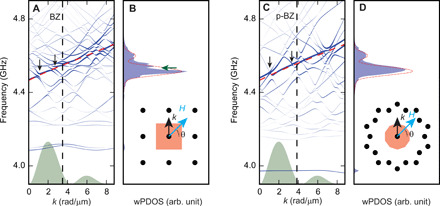
Band structures and DOS of the periodic and aperiodic lattice. (**A**) Band structure and (**B**) wPDOS of SWs of the SQ-MC. (**C**) Band structure and (**D**) wPDOS of SWs of the P3-MQC. In (A) and (C), the original SW dispersion relation of the YIG film is indicated by a red dashed line. Vertical arrows highlight bandgap openings. The size of blue dots in band structure graphs indicates the corresponding projection amplitudes ∣*P*_**G**_(*k*)∣ or ∣*P*_**F**_(*k*)∣, which display the spin precession amplitudes. In green, we show the excitation spectrum of the CPW microwave magnetic field in arbitrary units. A field value of 93 mT was assumed and applied at an angle of θ = 45°. Insets of (B) and (D) display reciprocal lattice points (i.e., end points of reciprocal lattice vectors **G** and reciprocal vectors **F**) and the BZ and p-BZ (orange areas) of SQ-MC and P3-MQC, respectively. The horizontal green arrow in (B) highlights the most prominent dip in the DOS induced by miniband formation. In (B) and (D), the wPDOS of SWs of the PF is indicated by a red dashed line.

Figure 6 displays SW band structures and wPDOS of SQ-MC and P3-MQC (blue curves) compared to the SW dispersion relation of the YIG film (red) at the field of the experiment applied with an angle of θ = 45°. The diameter of dots forming the blue curves in Fig. 6 (A and C) represents the projection amplitude of the SW eigenfunction ∣Ψ_***k***_⟩ onto a basis plane-wave mode ∣**G**⟩ (∣**F**⟩) corresponding to the reciprocal lattice vector **G** (reciprocal vector **F**) of the MC (MQC), relevant for backfolding wave vectors **k** into the first BZ (p-BZ) according to ∣*P*_**G**_(*k*)∣ = ∣〈**G**∣Ψ_**k**_〉∣(∣*P*_**F**_(*k*)∣ = ∣〈**F**∣Ψ_***k***_〉∣) (see Materials and Methods; fig. S3). The original SW dispersion relations (red dashed lines) fall onto a region with many backfolded modes in each of the two samples in Fig. 6 (A and C). At the angle of 45°, the group velocity of plane-film SWs is low. However, the numerous backfolded modes interact with the original dispersion relation and can have higher group velocities than the original mode. Such backfolded modes then carry the pronounced SW propagation signals observed experimentally in SQ-MC and P3-MQC (Fig. 4, E and H). At the same time, hybridization of modes occurs and novel allowed minibands form separated by small frequency gaps (highlighted by vertical arrows). Accordingly, the wPDOSs of SQ-MC and P3-MQC in Fig. 6 (B and D; light blue colored area) show fine structures of local maxima and dips (horizontal arrow), while the unstructured film (dashed red line) exhibits a smooth wPDOS. In fig. S4, we depict for direct comparison the band structures and DOS calculated at the four angles 0°, 35°, 45°, and 86° for SQ-MC and P3-MQC side by side. In the experiments, the absorption spectra do not feature all the predicted dips. We attribute this to SW damping in the real samples, which was not considered for the calculations.

## DISCUSSION

SWs in aperiodic P3-MQC are emitted quasi-omnidirectionally following the iso-frequency contour of the SW dispersion relation of the YIG film (fig. S2). For the periodic SQ-MC, we observe a notably different excitation pattern in *k*-space (fig. S2, C to E); here, the Fourier-transformed SW intensity displays resonance peaks at **k** ± **G**, which reside even outside the iso-frequency contour valid for an unpatterned film (yellow symbols in fig. S2, C to E). The SWs were imaged in the region where SWs propagated in the *y* direction, and therefore, we expected to see only FFT peaks with positive *k_y_* values. However, for SQ-MC, FFT peaks with negative *k_y_* values are also observed (Figs. 2E and 3, B and C). This indicates the formation of the Bloch waves of SWs in the periodically modulated MC. According to the dispersion relation of the YIG film, the intensity of the SW modes **k** + **G**_*i*(−1)_ (*i* = 0, ±1, ±2) is expected to be stronger than **k** + **G**_*i*1_ at *f* = 1.48, 1.62, and 1.8 GHz (fig. S5). However, the experimental results showed the opposite trend. This suggests that the coherent backscattering of SWs is modified by the grating. We assume the SW excitation at large absolute wave vectors to be allowed through the inhomogeneous broadening introduced by the unintentionally rough nanotroughs in the otherwise planar YIG film.

In (*34*), it was shown for periodically modulated films that the magnitude and width of forbidden gaps (dips) were tuned by the depth of nanotroughs. For the 1D surface-modulated MC of (*34*), the width of the first bandgap for DE-type SWs increased with the depth of stripe-like grooves and the correspondingly enhanced dipolar fields induced by dynamic magnetic surface charges. We thus speculate that by deeper nanotroughs in P3-MQC, more pronounced magnonic bandgap openings can be created. Complete bandgaps were realized for photonic quasicrystals. Here, the modification of individual elements induced a photonic waveguide (*35*). Along an irregular path, light was guided and bent at sharp corners (*36*). If complete bandgaps were reached for SWs, MQCs would form an analogous basis for SW waveguides. A large out-of-plane magnetic field was required in (*37*) to generate a complete bandgap in a periodic square lattice of antidots and exploit the isotropic forward volume waves propagating in a specific high-symmetry direction. Quasicrystals have, however, manifold rotational symmetries; therefore, complete bandgaps would allow one to create SW waveguides in a flexible manner and with a higher design degree of freedom concerning propagation directions.

In summary, we studied SW propagation and absorption in MQCs based on Penrose P3 tilings. We used BLS for phase-resolved SW microscopy and VNA for broadband SW spectroscopy. We obtained two important insights: (i) Omnidirectional SW emission in the MQC is observed by BLS microscopy and is attributed to the unconventional rotational symmetry of the Penrose P3 lattice; (ii) using broadband spectroscopy by means of VNA, we evidenced bandgap openings and correspondingly modified magnon DOS, consistent with analytical calculations. The unconventional symmetries are advantageous for integrated magnonic circuits in that optimized multidirectional magnon emission is realized at a single frequency and complete bandgaps might enable flexible magnon waveguides without applying a large out-of-plane magnetic field.

## MATERIALS AND METHODS

### Sample fabrication

The single-crystalline 100-nm-thick YIG films were grown on (111) GGG substrate by liquid phase epitaxy treated by Matesy GmbH (Jena, Germany). Al/Ti layers were evaporated on top of the YIG films with a thickness of 10/150 nm. The MCs and MQC masks were patterned on hydrogen silsesquioxane negative electron beam resists using electron beam lithography (EBL). Inductively coupled plasma etching and ion beam etching were performed to form a decagonal mesa and to introduce nanotroughs in the YIG film for the fabrication of the MCs and the MQC as summarized in Table 1. Samples were immersed into hydrofluoric acid bath to remove Al/Ti completely. Consecutively, CPWs were patterned on the MCs and MQC via EBL, and Ti/Au (4/200 nm) were sputtered before lift-off processes. Symmetry axes of SQ-MC and P3-MQC are parallel to the long axis of the CPWs. The width of a signal line and ground lines of the CPWs are 800 nm, and the gap between the signal and ground lines is 640 nm. A center-to-center separation between two CPWs is 12 μm.

Note that samples SQ-MC used in SW spectroscopy and BLS experiments are different. As indicated in Table 1, the diameters and depths of nanotroughs are different for each sample, while the characteristic length *a* stays the same. SQ-MC used for BLS measurement misses a column of nanotroughs indicated in fig. S6. As the missing column is far from the region of interest (ROI) for BLS experiment, we expect that the backfolding effect of the square lattice for SWs is still observed.

For the investigation of SW properties in quasicrystals, an identical P3-MQC was used. First, SW transport measurement was conducted by broadband SW spectroscopy technique. Afterward, one of the CPWs that used to be connected to port 2 of VNA was removed by injecting a large current. Then, spatial distribution of SWs was obtained by the BLS measurement. Note that a part of ROI is overlapped with the place where the CPW was mounted as shown in fig. S7.

### Broadband SW spectroscopy

SW excitations/propagations were studied via all-electrical SW spectroscopy. The two ends of the CPWs (patterned on top of the devices; see fig. S8) were connected to a VNA to apply microwaves. The in-plane angle θ of the applied field *H* was varied. A two-port VNA allowed us to generate a microwave magnetic field with frequencies ranging from 10 MHz to 26.5 GHz. The microwave with a power of −25 dBm was applied at the port 1(2) of the CPW to excite magnetization precession. The precession-induced voltage was detected at port 2 by reading the scattering parameter S_21_(S_22_), where the numbers 2 and 1(2) in the subscript denote the detection and excitation port. An external magnetic field μ_0_**H** of up to 90 mT was applied under an angle θ between the external field *H* and the CPW’s long axis. To increase signal-to-noise ratio, ΔS_21_ = S_21_(*H*) − S_21_(Ref) was evaluated, where S_21_(*H*) and S_21_(Ref) represent scattering parameters measured at a given field *H* and at 90 mT along θ = 90°, respectively.

### BLS microscopy

SW eigenmodes were imaged via BLS microscopy with or without phase resolution at room temperature using a setup similar to (*38*, *39*). Figure S9 shows a sketch of the experimental configuration. The end of the CPW was electrically bonded to a printed circuit board, and the circuit board was connected to a signal generator for a microwave application. The microwave excited spin precession in the MC/MQC near the CPW at a fixed frequency. A magnetic field of 10 mT was applied parallel to the long axis of the CPW via a permanent magnet for BLS measurement, and after that, a field of 90 mT was applied to first saturate the samples. A lens with a numerical aperture of 0.85 was used to focus a 473-nm wavelength laser with a spot size of 300 nm onto the samples. Laser power was set to 1 mW. Energy shifts of reflected laser light due to the inelastic magnon-photon scattering were detected by a triple-tandem Fabry-Pérot interferometer. The device was positioned under the laser spot using a *x*, *y* piezo-positioning system. The step size to acquire the SW images was 100 nm. We exploited phase-resolved inelastic light scattering while exciting SWs phase-coherently at the straight CPW. The nanotrough positions were reconstructed in that we overlaid (i) atomic force microscopy images taken on the studied MC/MQC, (ii) the BLS data, and (iii) the exposure masks for CPWs and EBL of nanotroughs.

### Bragg peaks of crystal/quasicrystal structure

Figure S10 (A and B) shows the square and Penrose P3 lattice used for the fabrication of the MC and MQC, respectively. The Penrose P3 tiling is composed of acute and obtuse rhombi structures with the same side length *a*, and the real space is filled out with these rhombi in a self-similar manner. The FFT of the crystal structure shows Bragg peaks as shown in fig. S10A. Bragg peaks of the square lattice correspond to its reciprocal lattice vectors **G***_ij_*, which are expressed as follows

$$G_{\mathrm{ij}}=iA+jB$$ (2)

where **A** = (2π/*a*,0) and **B** = (0,2π/*a*) and *i* and *j* are integer numbers. The lines bisecting the reciprocal lattice vectors **G**_10_, **G**_(−1)0_, **G**_01_, and **G**_0(−1)_ form the square-shaped BZ boundary in square-lattice based crystals as shown in fig. S10A.

In contrast to the periodic crystals, quasicrystals do not have reciprocal lattice vectors in *k*-space. A Bragg peak pattern is expressed with basic reciprocal vectors **C***_p_* = [2π/(*a* cos (π/10))] × ( cos (*p*π/5), sin (*p*π/5)), where *p* = 0,1,2,3,4. The reciprocal vectors are expressed by a linear combination of the basic reciprocal vectors

$$F=m_{0}C_{0}+m_{1}C_{1}+m_{2}C_{2}+m_{3}C_{3}+m_{4}C_{4}$$ (3)

which are chosen such that they (i) are related to the strongest Fourier coefficients describing the distribution of the material parameters in the quasicrystal, (ii) cover the reciprocal space with the appropriate density to describe the possible distribution of the magnon eigenmodes, and (iii) have the rotational symmetry of the diffraction pattern (*8*). *m_p_* are integer numbers. Here, $F_{q=p}^{\left(1\right)}=C_{p}$ and $F_{q=p+5}^{\left(1\right)}=-C_{p}$, where *q* = 0,1, …,9. Note that $F_{q+5}^{\left(1\right)}=-F_{q}^{\left(1\right)}$, and therefore, $F_{q+10}^{\left(1\right)}=F_{q}^{\left(1\right)}$. The other intense peaks are expressed as **F**^(0)^ = (0,0), $F_{q}^{\left(2\right)}=F_{q-1}^{\left(1\right)}+F_{q+2}^{\left(1\right)}$, and $F_{q}^{\left(3\right)}=F_{q}^{\left(1\right)}+F_{q+1}^{\left(1\right)}$, with the magnitude of ∣**F**^(0)^∣ = 0, ∣**F**^(2)^∣ = 2π/(*a* cos (π/5)), and ∣**F**^(3)^∣ = π/*a*, respectively. The intensity of **F**^(0)^, **F**^(2)^, and **F**^(3)^ in diffraction patterns of the Penrose P3 lattice is higher than the intensity of the basic reciprocal vectors **C***_p_*. Note that Bragg peaks of the Penrose P3–based quasicrystals exhibit 10-fold rotational symmetry. The lines bisecting the reciprocal vectors $F_{q}^{\left(1\right)}$ form the decagon-shaped p-BZ boundary in Penrose P3–based quasicrystals as shown in fig. S10B.

### Calculation of DOS for MC/MQC

To calculate the DOS of SW, the band structure of SWs in the MCs and MQCs needs to be calculated. Considering (*33*, *34*), the magnonic band structures for the MCs and MQCs are calculated using the plane wave method (*32*). For calculating the magnon bands, 49 smallest reciprocal lattice vectors, **G***_ij_*, with *i*, *j* = 0, ±1, ±2, ±3, are considered in the case of SQ-MC. In the case of P3-MQC, we considered 31 high-intensity Bragg peaks, **F**^(0)^ and $F_{q}^{\left(i\right)}$, with *i* = 1,2,3 and *q* = 0,1, …,9. The material and physical parameters used for the calculation are as follows: a saturation magnetization μ_0_*M_s_* = 180 mT, an exchange constant *A* = 3.65 × 10^−12^ J/m, a gyromagnetic ratio γ/2π = 28.024 GHz/T, characteristic length *a* = 900 nm, a diameter of nanotroughs *D* = 295 nm, a depth of nanotroughs *t* = 23 nm, and the thickness of the YIG film is 100 nm. The wave number of the first BZ (p-BZ) boundary of sample SQ-MC (P3-MQC) is 3.49 rad/μm (3.96 rad/μm) along the *k_y_* direction. An angular-dependent external field μ_0_*H*(θ) = (93 + 3 cos (2θ)) mT is considered for the analytic calculation to match the results with experimental data. The Fourier coefficients for circle defined in (*33*) are used for the calculation of magnonic band structures. The calculation result of the band structure for SQ-MC inside the first BZ at μ_0_*H* = 93 mT and θ = 45° is shown in fig. S3A. Eigenfrequencies *f*_η_(*k*) (η denotes the backfolded mode number) from this calculation display the band structure of the SQ-MC. Gap openings in an SW band structure of 1D MCs in DE configuration only appear at the BZ boundary. Here, 2D MCs even have partial bandgaps inside the BZ due to the hybridization of the backfolded branches of the BVW mode. Using the projection of the magnon eigenfunction, ∣Ψ_η,***k***_⟩, onto the plane wave mode ∣**G** > (∣**F**⟩), we calculated the PDOS of SWs for the MC (MQC) as follows

$$\rho_{G(F)}(f)=\frac{-1}{\pi }\sum_{\eta }\int dk\;\text{Im}[\frac{{|P_{\eta ,G(F)}(k)|}^{2}}{f-f_{\eta }(k)+i0^{+}}]$$ (4)

where 0^+^ is a positive infinitesimal value and *P*_η, **G**(**F**)_(*k*) = 〈**G**(**F**)∣Ψ_η,**k**_〉 is the projection amplitude. Depending on the MC or MQC, we either used **G** or **F**, respectively, in Eq. 4. η runs over the number of magnon bands (1 to 49 for MC and 1 to 31 for MQC). The limits of integration over *k* are 0 to 8.8 rad/μm. The size of blue dots in band structure graphs (see fig. S3) indicate the projection amplitude ∣*P*_η, *G*(*F*)_(*k*)∣, which corresponds to the spin precession amplitude. For the comparison to the SW spectra in reflection geometry using VNA, we consider the Fourier transformation of the excitation spectrum of the CPW-induced microwave magnetic field, *I*(*k*) (green transparent in Fig. 6A), as a weight factor and the wPDOS is expressed as

$$\rho_{G(F)}^{w}(f)=\frac{-1}{\pi }\sum_{\eta }\int dk\;\text{Im}[\frac{{|P_{\eta ,G(F)}(k)|}^{2}{|I(k)|}^{2}}{f-f_{\eta }(k)+i0^{+}}]$$ (5)

The range of the wave vectors covering the *k*_1_ and *k*_2_ modes of *I*(*k*) is up to 8.8 rad/μm, which is beyond the wave vector of the boundary of the first BZ of the periodic SQ-MC. To extend the dispersion relation beyond its first BZ (fig. S3), a magnon band structure with respect to the reciprocal lattice vectors **G** is considered, which are responsible for the backfolding of wave vectors **k** into the first BZ (vector **G**_01_ for our study; see fig. S3C). Then, the results are concatenated to the original band structure (Fig. 6A and fig. S3D). Figure 6B shows the calculated wPDOS of the SQ-MC at μ_0_*H* = 93 mT and θ = 45°.

For the aperiodic P3-MQC, we calculated the dispersion relations in that we considered wave vectors 0 ≤ *k* ≤ 8.8 rad/μm along the *k_y_* direction. The responsible reciprocal vector for the projection of magnon eigenfunctions was **F**^(0)^. Note that one cannot simply extend the dispersion relation of P3-MQC by further concatenating the band structure due to the aperiodicity. Figure 6C shows the dispersion relation of the P3-MQC at μ_0_*H* = 93 mT and θ = 45°. The most pronounced excitation around the *k*_1_ mode of the CPW (Fig. 4A) covers the p-BZ (vertical dashed line in Fig. 6C). The number of SW branches and characteristics of the gaps are different from the ones of the MC (Fig. 6A). The corresponding wPDOS is displayed in Fig. 6D. The calculations have been performed for different field orientation angles θ for SQ-MC and P3-MQC. The angular-dependent DOSs considering our experimental setting are shown in Fig. 5 (E and F).

## SUPPLEMENTARY MATERIALS

Supplementary material for this article is available at http://advances.sciencemag.org/cgi/content/full/7/35/eabg3771/DC1

## References

[1] D. Shechtman, The Discovery of Quasi-Periodic Materials, Nobel lecture, Stockholm, 8 December 2011, The Nobel Prize organisation.

[2] C. Nisoli, R. Moessner, P. Schiffer, Colloquium: Artificial spin ice: Designing and imaging magnetic frustration. Rev. Mod. Phys. 85, 1473–1490 (2013).

[3] Z. V. Vardeny, A. Nahata, A. Agrawal, Optics of photonic quasicrystals. Nat. Photonics 7, 177–187 (2013).

[4] T. Matsui, A. Agrawal, A. Nahata, Z. V. Vardeny, Transmission resonances through aperiodic arrays of subwavelength apertures. Nature 446, 517–521 (2007). 17392781 10.1038/nature05620

[5] L. Bursill, P. J. Lin, Penrose tiling observed in a quasi-crystal. Nature 316, 50–51 (1985).

[6] A. L. Mackay, Crystallography and the Penrose pattern. Phys. A Stat. Mech. Appl. 114, 609–613 (1982).

[7] M. Bayindir, E. Cubukcu, I. Bulu, E. Ozbay, Photonic band gaps and localization in two-dimensional metallic quasicrystals. Europhys. Lett. 56, 41–46 (2001).

[8] M. A. Kaliteevski, S. Brand, R. A. Abram, T. F. Krauss, R. D. Rue, P. Millar, Two-dimensional Penrose-tiled photonic quasicrystals: From diffraction pattern to band structure. Nanotechnology 11, 274–280 (2000).

[9] M. C. Rechtsman, H.-C. Jeong, P. M. Chaikin, S. Torquato, P. J. Steinhardt, Optimized structures for photonic quasicrystals. Phys. Rev. Lett. 101, 073902 (2008). 18764536 10.1103/PhysRevLett.101.073902

[10] V. S. Bhat, J. Sklenar, B. Farmer, J. Woods, J. T. Hastings, S. J. Lee, J. B. Ketterson, L. E. De Long, Controlled magnetic reversal in permalloy films patterned into artificial quasicrystals. Phys. Rev. Lett. 111, 077201 (2013). 23992078 10.1103/PhysRevLett.111.077201

[11] J. Rychły, J. W. Kłos, M. Mruczkiewicz, M. Krawczyk, Spin waves in one-dimensional bicomponent magnonic quasicrystals. Phys. Rev. B 92, 054414 (2015).

[12] S. Watanabe, V. S. Bhat, K. Baumgaertl, D. Grundler, Direct observation of worm-like nanochannels and emergent magnon motifs in artificial ferromagnetic quasicrystals. Adv. Funct. Mater. 30, 2001388 (2020).

[13] J. Rychły, S. Mieszczak, J. W. Kłos, Spin waves in planar quasicrystal of penrose tiling. J. Magn. Magn. Mater. 450, 18–23 (2018).

[14] F. Lisiecki, J. Rychły, P. Kuświk, H. Głowiński, J. W. Kłos, F. Groß, I. Bykova, M. Weigand, M. Zelent, E. J. Goering, G. Schütz, G. Gubbiotti, M. Krawczyk, F. Stobiecki, J. Dubowik, J. Gräfe, Reprogrammability and scalability of magnonic Fibonacci quasicrystals. Phys. Rev. Appl. 11, 054003 (2019).

[15] F. Lisiecki, J. Rychły, P. Kuświk, H. Głowiński, J. W. Kłos, F. Groß, N. Träger, I. Bykova, M. Weigand, M. Zelent, E. J. Goering, G. Schütz, M. Krawczyk, F. Stobiecki, J. Dubowik, J. Gräfe, Magnons in a quasicrystal: Propagation, extinction, and localization of spin waves in Fibonacci structures. Phys. Rev. Appl. 11, 054061 (2019).

[16] V. S. Bhat, B. Farmer, N. Smith, E. Teipel, J. Woods, J. Sklenar, J. B. Ketterson, J. T. Hastings, L. D. Long, Non-stochastic switching and emergence of magnetic vortices in artificial quasicrystal spin ice. Phys. C: Superconduct. Appl. 503, 170–174 (2014).

[17] V. V. Kruglyak, S. O. Demokritov, D. Grundler, Magnonics. J. Phys. D Appl. Phys. 43, 264001 (2010).

[18] A. Khitun, M. Bao, K. L. Wang, Magnonic logic circuits. J. Phys. D Appl. Phys. 43, 264005 (2010).

[19] Y. Akahane, T. Asano, B.-S. Song, S. Noda, High-q photonic nanocavity in a two-dimensional photonic crystal. Nature 425, 944–947 (2003). 14586465 10.1038/nature02063

[20] S. Mamica, M. Krawczyk, D. Grundler, Nonuniform spin-wave softening in two-dimensional magnonic crystals as a tool for opening omnidirectional magnonic band gaps. Phys. Rev. Appl. 11, 054011 (2019).

[21] H. Yu, G. Duerr, R. Huber, M. Bahr, T. Schwarze, F. Brandl, D. Grundler, Omnidirectional spin-wave nanograting coupler. Nat. Commun. 4, 2702 (2013). 24189978 10.1038/ncomms3702 PMC3831280

[22] H. Yu, O. d’ Kelly, V. Cros, R. Bernard, P. Bortolotti, A. Anane, F. Brandl, F. Heimbach, D. Grundler, Approaching soft x-ray wavelengths in nanomagnet-based microwave technology. Nat. Commun. 7, 11255 (2016). 27063401 10.1038/ncomms11255 PMC4831022

[23] S. Maendl, D. Grundler, Multi-directional emission and detection of spin waves propagating in yttrium iron garnet with wavelengths down to about 100 nm. Appl. Phys. Lett. 112, 192410 (2018).

[24] K. Baumgaertl, J. Gräfe, P. Che, A. Mucchietto, J. Förster, N. Träger, M. Bechtel, M. Weigand, G. Schütz, D. Grundler, Nanoimaging of ultrashort magnon emission by ferromagnetic grating couplers at GHz frequencies. Nano Lett. 20, 7281–7286 (2020). 32830984 10.1021/acs.nanolett.0c02645 PMC7564445

[25] H. Yu, O. d’ Kelly, V. Cros, R. Bernard, P. Bortolotti, A. Anane, F. Brandl, R. Huber, I. Stasinopoulos, D. Grundler, Magnetic thin-film insulator with ultra-low spin wave damping for coherent nanomagnonics. Sci. Rep. 4, 6848 (2014). 25355200 10.1038/srep06848 PMC4213793

[26] T. Schwarze, D. Grundler, Magnonic crystal wave guide with large spin-wave propagation velocity in CoFeB. Appl. Phys. Lett. 102, 222412 (2013).

[27] R. Zivieri, S. Tacchi, F. Montoncello, L. Giovannini, F. Nizzoli, M. Madami, G. Gubbiotti, G. Carlotti, S. Neusser, G. Duerr, D. Grundler, Bragg diffraction of spin waves from a two-dimensional antidot lattice. Phys. Rev. B 85, 012403 (2012).

[28] S. Neusser, G. Duerr, S. Tacchi, M. Madami, M. L. Sokolovskyy, G. Gubbiotti, M. Krawczyk, D. Grundler, Magnonic minibands in antidot lattices with large spin-wave propagation velocities. Phys. Rev. B 84, 094454 (2011).

[29] S. Neusser, G. Duerr, H. G. Bauer, S. Tacchi, M. Madami, G. Woltersdorf, G. Gubbiotti, C. H. Back, D. Grundler, Anisotropic propagation and damping of spin waves in a nanopatterned antidot lattice. Phys. Rev. Lett. 105, 067208 (2010). 20868008 10.1103/PhysRevLett.105.067208

[30] B. A. Kalinikos, A. N. Slavin, Theory of dipole-exchange spin wave spectrum for ferromagnetic films with mixed exchange boundary conditions. J. Phys. C Solid State Phys. 19, 7013–7033 (1986).

[31] J. H. Kwon, J. Yoon, P. Deorani, J. M. Lee, J. Sinha, K.-J. Lee, M. Hayashi, H. Yang, Giant nonreciprocal emission of spin waves in Ta/Py bilayers. Sci. Adv. 2, e1501892 (2016). 27419231 10.1126/sciadv.1501892 PMC4942323

[32] M. Krawczyk, H. Puszkarski, Plane-wave theory of three-dimensional magnonic crystals. Phys. Rev. B 77, 054437 (2008).

[33] R. A. Gallardo, A. Banholzer, K. Wagner, M. Körner, K. Lenz, M. Farle, J. Lindner, J. Fassbender, P. Landeros, Splitting of spin-wave modes in thin films with arrays of periodic perturbations: Theory and experiment. New J. Phys. 16, 023015 (2014).

[34] R. A. Gallardo, T. Schneider, A. Roldán-Molina, M. Langer, J. Fassbender, K. Lenz, J. Lindner, P. Landeros, Dipolar interaction induced band gaps and flat modes in surface-modulated magnonic crystals. Phys. Rev. B 97, 144405 (2018).

[35] C. Jin, B. Cheng, B. Man, Z. Li, D. Zhang, S. Ban, B. Sun, Band gap and wave guiding effect in a quasiperiodic photonic crystal. Appl. Phys. Lett. 75, 1848–1850 (1999).

[36] Y. An, Z. Gao, Z. Ouyang, Surface wave photonic quasicrystal. Appl. Phys. Lett. 116, 151104 (2020).

[37] T. Schwarze, R. Huber, G. Duerr, D. Grundler, Complete band gaps for magnetostatic forward volume waves in a two-dimensional magnonic crystal. Phys. Rev. B 85, 134448 (2012).

[38] V. E. Demidov, S. O. Demokritov, Magnonic waveguides studied by microfocus Brillouin light scattering. IEEE Trans. Mag. 51, 0800215 (2015).

[39] T. Sebastian, K. Schultheiss, B. Obry, B. Hillebrands, H. Schultheiss, Micro-focused Brillouin light scattering: Imaging spin waves at the nanoscale. Front. Phys. 3, 35 (2015).

